# Identification of Chromatin-Associated Regulators of MSL Complex Targeting in *Drosophila* Dosage Compensation

**DOI:** 10.1371/journal.pgen.1002830

**Published:** 2012-07-26

**Authors:** Erica Larschan, Marcela M. L. Soruco, Ok-Kyung Lee, Shouyong Peng, Eric Bishop, Jessica Chery, Karen Goebel, Jessica Feng, Peter J. Park, Mitzi I. Kuroda

**Affiliations:** 1 Department of Molecular Biology, Cellular Biology, and Biochemistry, Brown University, Providence, Rhode Island, United States of America; 2 Division of Genetics, Department of Medicine, Brigham and Women's Hospital, Boston, Massachusetts, United States of America; 3 Department of Genetics, Harvard Medical School, Boston, Massachusetts, United States of America; 4 Center for Biomedical Informatics, Harvard Medical School, Boston, Massachusetts, United States of America; Institute for Molecular Biology and Tumour Research, Germany

## Abstract

Sex chromosome dosage compensation in *Drosophila* provides a model for understanding how chromatin organization can modulate coordinate gene regulation. Male *Drosophila* increase the transcript levels of genes on the single male X approximately two-fold to equal the gene expression in females, which have two X-chromosomes. Dosage compensation is mediated by the Male-Specific Lethal (MSL) histone acetyltransferase complex. Five core components of the MSL complex were identified by genetic screens for genes that are specifically required for male viability and are dispensable for females. However, because dosage compensation must interface with the general transcriptional machinery, it is likely that identifying additional regulators that are not strictly male-specific will be key to understanding the process at a mechanistic level. Such regulators would not have been recovered from previous male-specific lethal screening strategies. Therefore, we have performed a cell culture-based, genome-wide RNAi screen to search for factors required for MSL targeting or function. Here we focus on the discovery of proteins that function to promote MSL complex recruitment to “chromatin entry sites,” which are proposed to be the initial sites of MSL targeting. We find that components of the NSL (Non-specific lethal) complex, and a previously unstudied zinc-finger protein, facilitate MSL targeting and display a striking enrichment at MSL entry sites. Identification of these factors provides new insight into how MSL complex establishes the specialized hyperactive chromatin required for dosage compensation in *Drosophila*.

## Author Summary

Gene regulation is essential to all living things. For example, levels of gene expression in individual cells must be fine-tuned during development and in response to changing environmental conditions. Genes are regulated by DNA binding proteins and by factors that influence DNA packaging into chromatin. The MSL complex in *Drosophila melanogaster* is a chromatin-modifying complex that specifically regulates a large number of genes. The MSL complex targets active genes on the single male X chromosome to upregulate their output to match both female X chromosomes. How the MSL complex specifically targets the X chromosome and upregulates active genes is only partially understood. In order to increase our understanding of gene regulation at a mechanistic level, we performed a genome-wide genetic screen in male cells to identify factors that facilitate MSL targeting and function. Our results identify two chromatin-associated protein complexes and a new candidate DNA binding protein as key factors in MSL–based regulation. We also provide an extensive list of additional candidate genes to be examined in future studies.

## Introduction

X-chromosome dosage compensation in male *Drosophila* provides a model for understanding how a large number of diversely regulated genes along the length of a single chromosome can be targeted for coordinate regulation. In *Drosophila*, the transcript levels of genes along the length of the single male X-chromosome are upregulated approximately two-fold, a process mediated by the MSL (Male-specific lethal) histone acetyltransferase complex [1]. A model, initially based on genetic observations [2], posits that the MSL complex binds the X-chromosome in a two-step process. First, high levels of the MSL complex accumulate at approximately 150–300 “chromatin entry sites” (CES) containing GA-rich MRE (MSL Recognition Element) sequences that are ∼two-fold enriched on the male X [3] [4]. Second, a sequence-independent spreading occurs in which the MSL complex associates with the bodies of active X-linked genes via general features associated with transcription, including H3K36me3 [5]. MSL complex increases transcript levels of its target genes by increasing the density of RNA Polymerase II (RNAP II) over transcription units, likely via MSL-dependent H4K16 acetylation [6].

While significant progress has been made towards understanding how MSL complex is targeted and functions, much remains to be understood. For example, since MREs are less than two-fold enriched on the X-chromosome [3] and the core MSL complex does not appear to have sequence-specific DNA binding activity [7], it is likely that additional factors are involved in the key process of CES recognition.

Seminal genetic screens identified five core components of the MSL complex by isolating genes that were specifically required for male viability [8] [9]. However, these approaches would not recover a potentially key class of regulators that might be required for viability in both sexes, and act in dosage compensation by providing an interface with the core transcriptional machinery. In fact, based on experiments from budding yeast [10] [11] the essential SET2 H3K36 methyltransferase was identified as a potential regulator of MSL complex association with active genes [5] [12]. SET2 contributes to targeting of the MSL complex to the bodies of active genes [5] [12], but it is likely that other factors also participate. Furthermore, there are no candidates for direct binding to the MRE sequence, which is proposed to be a key first step in MSL targeting.

Therefore, we designed a cell-based reporter system that specifically monitors MSL function at chromatin entry sites, and conducted a genome-wide RNAi screen to identify genes required to regulate this reporter. We identified the NSL [13] [14] and PAF [15] [16] general transcriptional regulators, as well as a previously unstudied zinc finger protein, CG1832, that emerges as a strong candidate to function as the previously unknown link between MSL complex and MRE sequences within chromatin entry sites.

## Results

### A genome-wide RNAi screen identifies genes that regulate an MSL-dependent reporter in male SL2 cells

**Figure 1:**
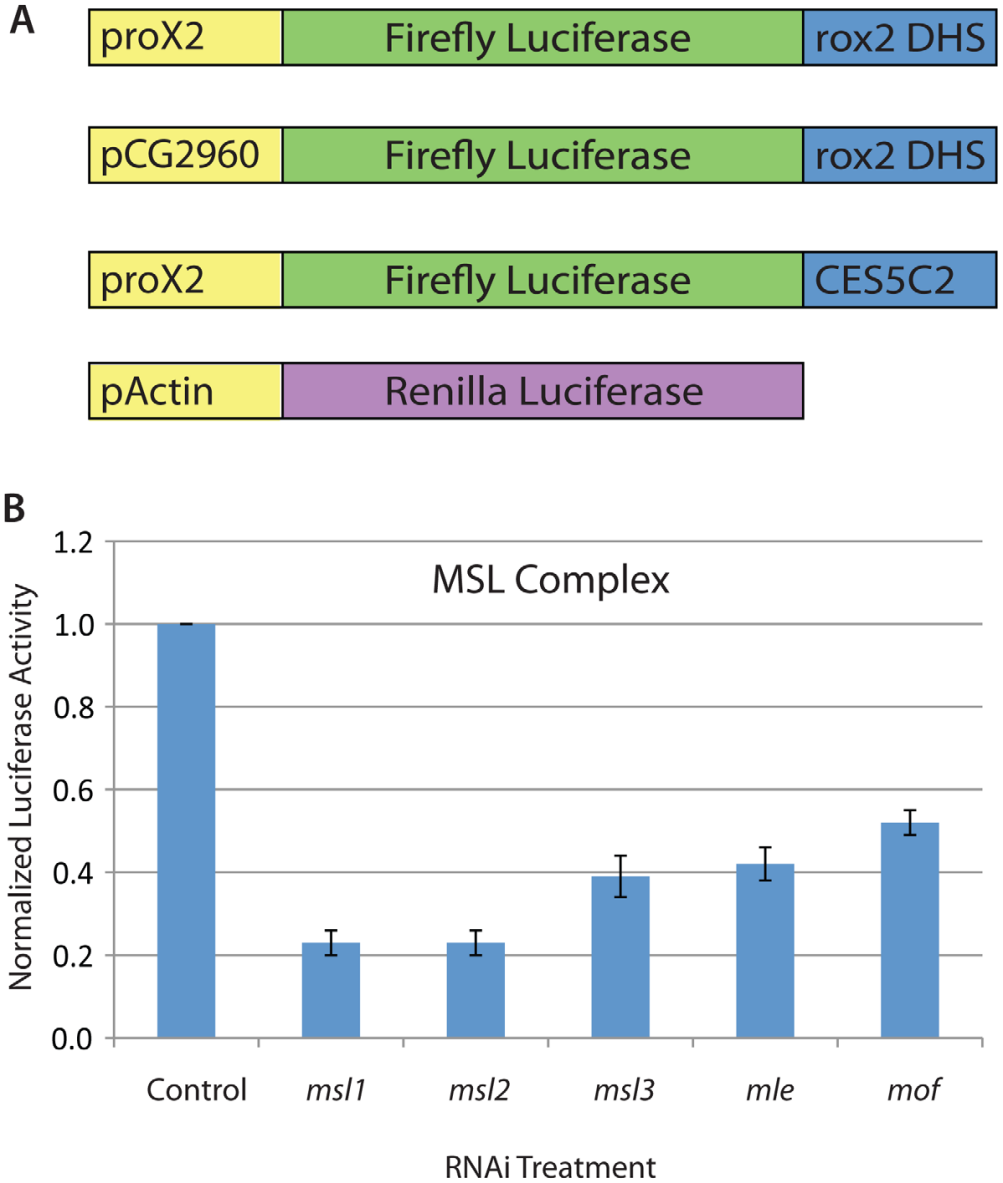
An RNAi screening system in *Drosophila* SL2 cells to identify regulators of MSL complex recruitment and function. A) Schematic of the MSL-dependent reporter system for SL2 cell-based genome-wide RNAi screening. The system includes the following three elements: 1) promoter of *roX2* gene (370 bps); 2) Firefly luciferase reporter gene; 3) *roX2* DHS (DNase I Hypersensitivity Site: 280 bp). Two additional constructs were used for secondary screening in which the promoter or CES were altered. The alternative promoter is that of the CG2690 gene and the alternative CES is the minimal 150 bp fragment of CES5C2 that were previously described [3]. B) Ratio of the Firefly luciferase activity to Renilla luciferase activity for MSL complex component RNAi treatments identified from the genome-wide screen. Data shown are the average and standard deviation of two replicates from the genome-wide screening plates. Control RNAi treatment targeted the GFP gene that is not present in SL2 cells.

In order to identify genes that regulate MSL complex recruitment or function, we performed a genome-wide RNAi screen using an MSL-dependent cell-based reporter system for which we previously reported functionality [3] (Figure 1A). This reporter system includes the following three elements: 1) promoter of *roX2* gene (370 bp); 2) Firefly luciferase reporter gene; 3) MSL complex binding site for roX2 (DNase I Hypersensitivity Site: 280 bp). The system is based on a similar one that functions as a male-specific MSL-dependent reporter *in vivo* [17]. A genome-wide RNAi library generated by the *Drosophila* RNAi Screening Center (DRSC) that contains approximately 21,000 dsRNAs was screened in duplicate (www.flyrnai.org). To eliminate off-target effects, secondary screens were conducted using a set of validation RNAi constructs in addition to rescreening the original constructs.

In addition to direct regulators of MSL complex recruitment or function, several additional types of genes could be identified by our RNAi screen. Therefore, we used the following steps to identify potential direct activators of MSL complex targeting: 1) To reduce the number of non-specific regulators of luciferase gene transcription and protein stability that were identified, we calculated the ratio of activity from our MSL-dependent Firefly luciferase reporter to a heterologous Renilla luciferase reporter. 2) We used multiple MSL-dependent reporters to further narrow the list of genes to those that function independently of the *roX2* promoter and specific CES used in the genome-wide screen; 3) We then used known functional information about the genes to narrow the list to those that have potential direct activating roles; 4) We determined whether new proteins affect MSL complex recruitment at endogenous sites without altering transcription of MSL complex components; 5) We performed genome-wide localization analysis to determine whether new proteins are enriched at CES loci.

As briefly described above, we first normalized the MSL-dependent Firefly luciferase reporter activity to an MSL-independent reporter including the Renilla luciferase gene that can be independently assayed using a different substrate. This dual Firefly/Renilla approach also controls for technical variation in transfection efficiencies among wells and differences in cell number caused by altered viability after RNAi treatments. We used a Renilla construct that was regulated by an Actin promoter as previously described [18]. Therefore, by calculating the ratio of Firefly/Renilla luciferase activity, RNAi conditions that specifically alter the MSL-dependent reporter but not the Actin-Renilla construct could be identified.

To conduct the RNAi screen, sixty-two screening plates containing approximately 21,000 dsRNAs arrayed in individual wells of a 384-well plate were assayed in duplicate. Each plate contained two positive control (*msl2* RNAi) and two negative control (GFP RNAi) constructs, that were previously validated. After confirming that at least a 5-fold dynamic range was observed in the expected activity of positive and negative controls on each plate, the Firefly/Renilla ratios of the two plates were averaged and screening hits were identified relative to the median Firefly/Renilla ratio for each plate (See Methods). We also conducted the same computational analysis for the Renilla values and removed hits in which the Renilla activity was dramatically altered by the RNAi treatment (See Methods).

Our screening approach identified 322 RNAi constructs that altered MSL-dependent reporter activity (Table S1). Importantly, we found all known components of the MSL complex in an unbiased way, validating our screen (Figure 1B). We identified 254 RNAi constructs that reduced MSL-dependent reporter activity and 68 RNAi constructs that increased reporter activity. Gene Ontology analysis revealed several functional categories that were enriched including the following: protein synthesis, cell cycle control, and transcriptional regulation (p<0.05) (Table 1). Similar analysis of publically available data sets from other RNAi screens performed using the same library indicated that the transcriptional regulation Gene Ontology category was not enriched in any other publically available screens. In contrast, the protein synthesis category and cell cycle control categories were commonly enriched in the majority of screens conducted with this RNAi library.

**Table 1:** Gene ontology categories that were enriched in the genome-wide RNAi screen.

Functional Category	P-value
Protein biosynthesis	1.01E-22
Cell cycle	4.24E-07
Cell cycle control	1.18E-06
mRNA transcription	4.03E-06
Protein metabolism and modification	3.38E-05
Nucleoside, nucleotide and nucleic acid metabolism	1.03E-04
mRNA transcription regulation	2.99E-02

Next, candidate genes were chosen for further analysis and validation with additional RNAi constructs. Three classes of genes were removed from the original list of candidates: 1) Ribosomal protein genes that are identified by many RNAi screens (41 genes); 2) Genes with a large number of predicted RNAi off targets using 17 bp as an overlap cutoff [19] (19 genes) 3) RNAi amplicons that did not target a specific gene and/or target multiple predicted genes (21 amplicons). We rescreened the 241 remaining candidates using both original and validation RNAi constructs with the original screening system and two additional MSL-dependent reporters that we previously validated [3]. These additional MSL-dependent reporters had a different promoter or CES compared with the original RNAi screening construct and were previously described (Figure 1A). In this way, we identified 72 genes that were validated with an additional RNAi construct and regulated all three MSL-dependent reporters (Table S2). We do not wish to over-interpret any negative results; however, we did not identify the genes encoding HP1, SU(VAR)3–7, or ISWI. These each have a mutant phenotype that differentially affects the polytene X chromosome [20] [21], but may not affect MSL function. Likewise, we did not identify JIL-1, SCF, NUP153, or Megator, all previously implicated in MSL interaction [22] [23] [13].

To narrow our list of candidate genes, we classified genes into categories based on their activity in our assay and previously determined functional information about the gene products as follows (Table S2): 1) **Class 1 (18 genes):** General transcriptional regulators that *activate* our reporter; 2) **Class 2 (6 genes):** Proteins that have potential sequence-specific DNA binding activity and *activate* our reporter; 3) **Class 3 (7 genes):** General transcriptional regulators that *repress* our reporter; 4) **Class 4 (3 genes):** Proteins that have potential sequence-specific DNA binding activity and *repress* our reporter; 5) **Class 5 (7 genes):** Proteins that regulate RNA metabolism (e.g. splicing, non-sense mediated decay); 6) **Class 6 (26 genes):** Proteins that are previously unstudied and/or have functions unrelated to transcription or RNA metabolism. The remaining genes are known MSL complex components. Members of each of these classes could directly modulate MSL recruitment or activity or indirectly effect MSL complex by altering complex levels.

**Figure 2:**
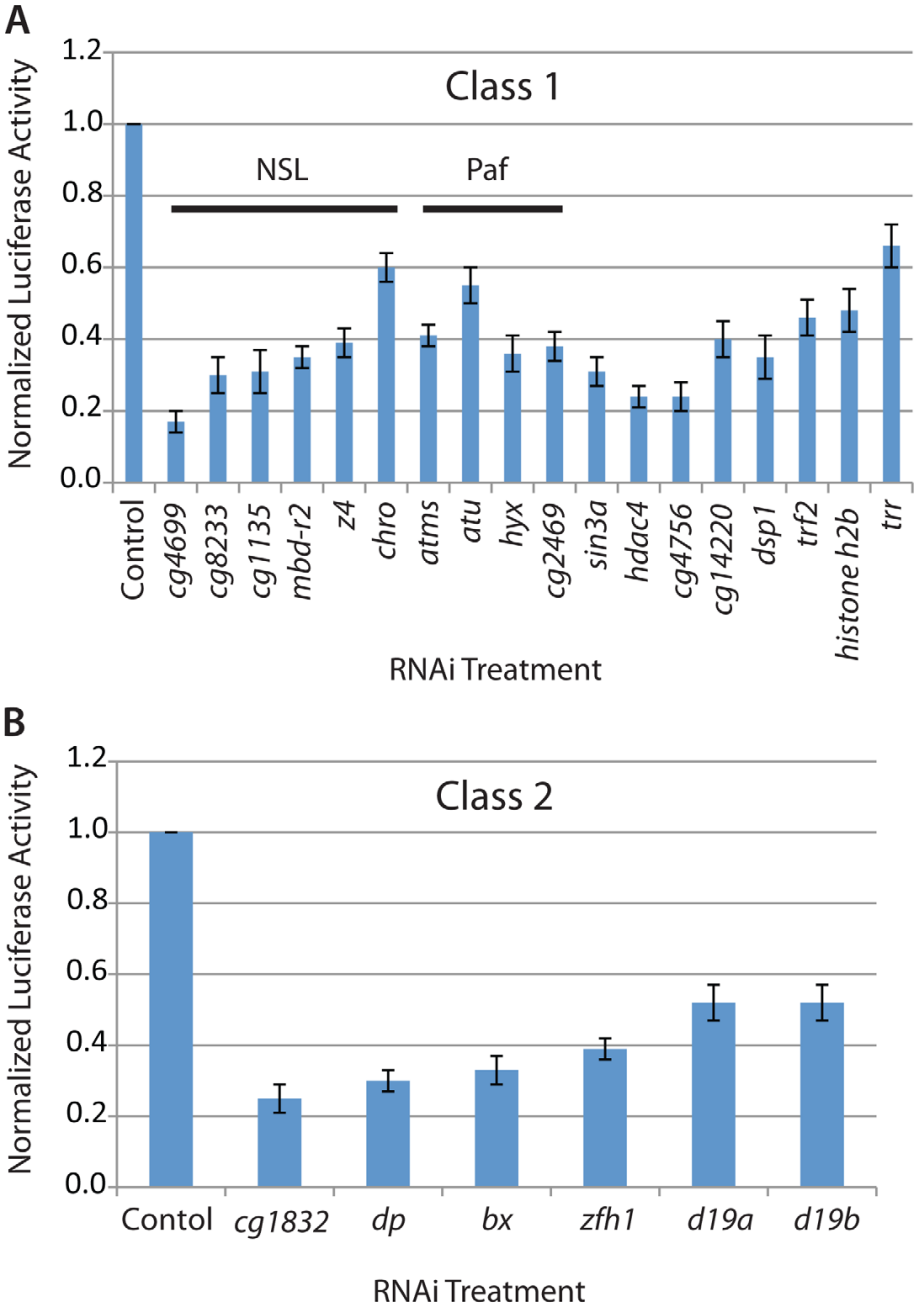
Candidate regulators of MSL complex identified from the genome-wide RNAi screen. The Firefly/Renilla luciferase activity ratio and its standard deviation was determined based on quadruplicate reactions in 96-well plate format after RNAi treatment targeting the following classes of candidate genes: A) Class 1: NSL complex, PAF complex and other general transcriptional regulators; B) Class 2: Activators with potential specific DNA binding activity like CG1832.

Due to the large number of genes identified, we focused on potential direct regulators of MSL complex recruitment or function that had known roles in transcriptional regulation or potential DNA binding domains (Classes 1–4). This analysis identified 34 potential transcriptional regulators. We validated the activity of these candidate genes using our MSL-dependent reporter assay in a more accurate 96-well format (Figure 2). Overall, we identified 24 activators (Classes 1 and 2) and 9 repressors (Classes 3 and 4) with known or predicted roles in transcriptional regulation.

In addition to identifying all core components of MSL complex as activators of our reporter system (Figure 1B), we identified a known repressor of MSL function, NURF301, which we previously determined represses *roX* gene transcription *in vivo* [24] (Table S2). Also, NURF301 is known to associate with the *roX1* CES [24]. Therefore, our RNAi screening strategy identified all MSL complex components and a known direct repressor of MSL-dependent transcription, validating our ability to identify activators and repressors with direct functions.

### Selection of NSL complex, PAF complex, and CG1832 as candidate direct regulators of MSL targeting or function

The transcription of MSL complex components could be modulated and thus indirectly affect its function. Therefore, we selected candidates and examined whether they function directly to recruit MSL complex to CES loci using several approaches: 1) Determine whether RNAi against candidate genes affects recruitment of MSL complex to endogenous CES; 2) Define whether RNAi knockdown of candidates alters transcription of MSL complex components; 3) Examine the recruitment of candidate proteins to CES loci. We hypothesized that direct regulators of MSL complex recruitment will be recruited to CES loci and alter MSL complex recruitment but not transcription of MSL complex components. Using these approaches, we examined the function of three factors: 1) the NSL complex; 2) the PAF complex; and 3) CG1832.

We selected NSL complex for further study because six of its components (NSL1, NSL3, CG1135, z4, Chro, and MBD-R2) were identified in our screen and NSL and MSL complexes share the MOF catalytic component. NSL1 is one of the structural components of NSL complex that directly contacts MOF using a similar protein-protein interaction surface as MSL1 [25]. Although this biochemical relationship would predict that NSL complex would compete with MSL complex for the MOF subunit, NSL complex was identified as an activator of our MSL-dependent reporter. Therefore, we further characterized the relationship between the two complexes to determine whether NSL complex functions positively during MSL complex recruitment.

We also examined the PAF transcription complex that associates with RNAP II across gene bodies to promote transcription elongation [26] [16]. Again, we identified multiple subunits of this complex in our screen, making the PAF complex a strong candidate. The PAF complex has been implicated in facilitating H2B ubquitylation catalyzed by the *S. cerevisiae* SAGA complex component Ubp8 [27] [28]. Intriguingly, recent work has identified a new activity for the MSL2 protein as an E3 ligase that targets H2BK34 for ubiquitylation (H2BK31 in *Drosophila*) [29]. Therefore, it is possible that the PAF complex facilitates the H2B ubiquitin ligase activity of MSL2. Also, MSL complex associates with gene bodies of active genes like the PAF complex [30] [31].

The previously-unstudied CG1832 zinc finger protein was chosen for further study because it was identified three independent times using different double-stranded RNA constructs and exhibits a very strong reporter activation phenotype. Furthermore, unlike other potential DNA binding proteins, CG1832 RNAi does not decrease cell viability significantly and was not identified by other RNAi screens. Similarly, loss of MSL complex components does not significantly alter cell viability although it causes male-specific lethality in flies. The CG1832 protein has a glutamine rich N-terminus and a seven zinc finger domain at its C-terminus. Because each finger is likely to recognize three bases [32], CG1832 is a candidate for recognition of a 21-mer sequence such as the MRE element. CG1832 is highly conserved among insect species and orthologs are present in other species such as mouse (znf80) and human (znf429). Furthermore, CG1832 is maternally loaded into the early embryo and ubiquitiously expressed (FlyAtlas) and therefore it could potentially target MSL complex early in development. In addition, CG1832 is likely to be an essential gene because P-element insertions at its 5′ end are lethal in both sexes (data not shown). Therefore, CG1832 would not have been identified by the seminal male-specific lethal screens *in vivo*.

### CG1832 and NSL complex modulate MSL complex recruitment to endogenous CES loci *in vivo*

**Figure 3:**
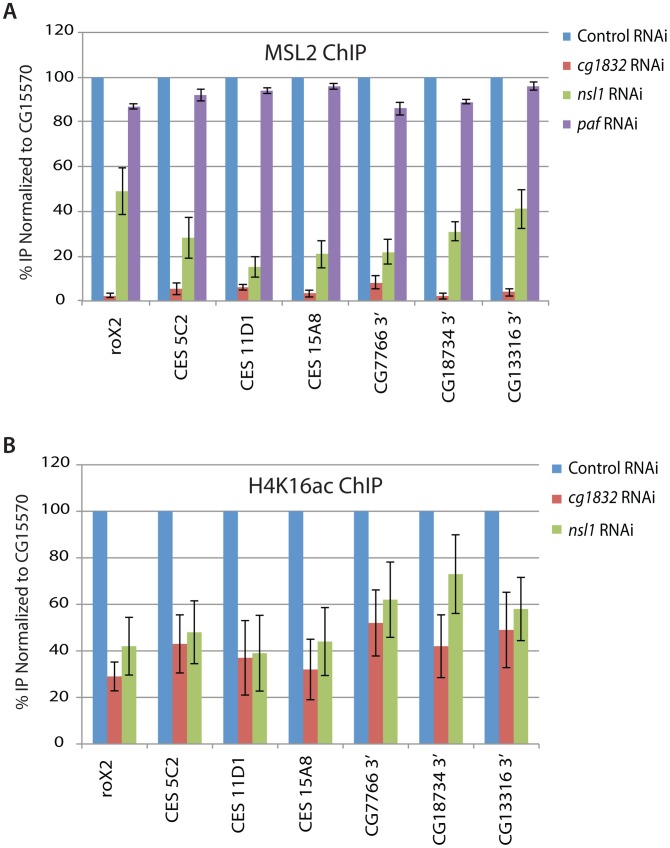
NSL1 and CG1832 contribute to MSL complex recruitment. A) Chromatin immunoprecipitation was conducted using an antibody specific for the MSL2 protein before and after RNAi treatment targeting the *paf1*, *nsl1* and *CG1832* genes. Four CES loci and the 3′ ends of three previously characterized MSL complex target genes were assayed [3], [5]. Data shown are the average and standard deviation of at least two replicates. Normalization was conducted both to input DNA material and to the non-transcribed CG15570 gene as an internal control as previously described [42]. B) Chromatin immunoprecipitation was conducted as described for (A) but an antibody targeting the H4K16ac histone modification (Millipore) was used instead of the MSL2 antibody. In addition to the normalization as described for (A), H4K16ac IPs were normalized to histone H3 occupancy as determined by H3 ChIP. Due to significant changes in histone H3 occupancy upon *paf1* RNAi treatment, these data were not included.

To determine whether candidate genes are needed for MSL complex recruitment to endogenous CES loci, we used chromatin immunoprecipitation (ChIP) of the MSL2 core component in male SL2 cells. We found that both NSL1 and CG1832 RNAi knockdowns significantly reduce MSL2 recruitment to several CES loci *in vivo* (Figure 3A). Next, we conducted ChIP on the H4K16ac histone modification to examine the roles of NSL1 and CG1832 in depositing this key modulator of dosage compensation. In parallel with the effect on MSL2 recruitment, both NSL1 and CG1832 RNAi caused reduced H4K16ac at several CES loci. In contrast, PAF RNAi treatment did not alter MSL complex recruitment and therefore it is likely that PAF functions to alter MSL complex function at a step subsequent to recruitment (Figure 3A).

To examine whether NSL1 and CG1832 RNAi treatments disrupt MSL complex recruitment due to regulation of MSL complex component mRNA levels, we assayed these by qRT-PCR (Figure S1). *roX1* is not expressed at significant levels in SL2 cells [33]. We noted effects on *roX2* RNA levels as expected because *roX2* is activated by MSL complex, but little effect on the mRNA levels of other MSL complex components. Also, NSL1 and CG1832 RNAi treatments do not affect mRNA splicing of MLE as reported for the Znf72D zinc finger protein (data not shown) [34]. MLE splicing was quantified using splice-site specific primers that were previously validated [34]. Because improper targeting of MSL complex reduces complex stability [35], precise measurements of MSL protein levels do not address the point at which NSL1 or CG1832 acts to promote MSL complex activity. Instead, we tested the hypothesis that these proteins promote MSL recruitment by assessing their own localization to CES loci.

### Genome-wide occupancy of NSL components and CG1832 support a direct role in MSL targeting

**Figure 4:**
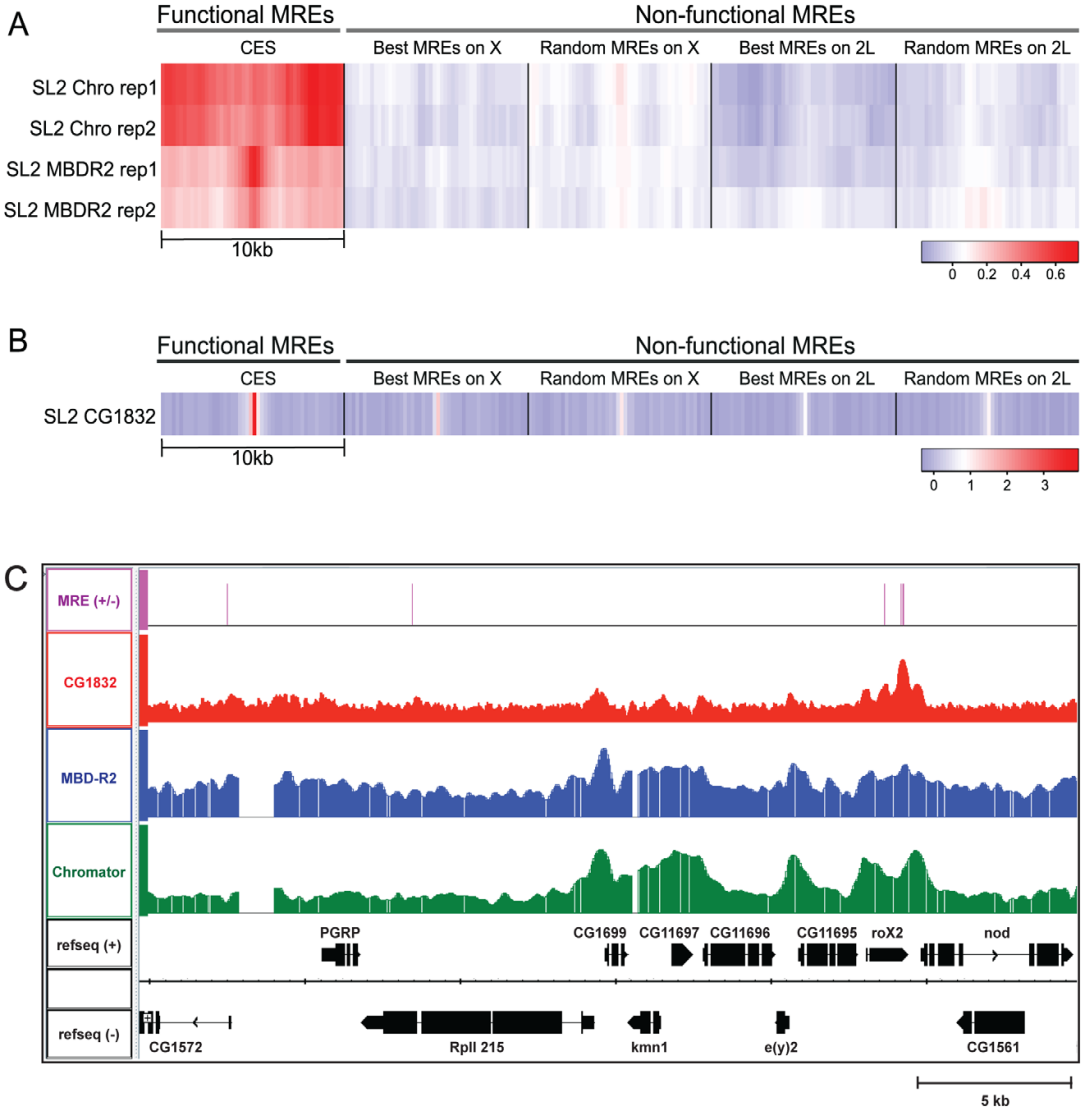
NSL complex and CG1832 enrichment at CES loci. A) Heatmaps show the average enrichment of the Chromator/Chriz and MBD-R2 components of the NSL complex surrounding MRE sequences as assayed by ChIP-chip in SL2 cells (two replicates each; data available from http://www.modENCODE.org). The darker red color indicates greater occupancy, as measured in the log2 (ChIP/input) scale. Heatmaps are shown for sets of 150 sites containing MREs that are defined as follows: MREs in chromatin entry sites (column 1); best MREs on the X chromosome or autosome 2L (MRE motifs closest to the consensus, but not utilized, columns 2 and 4); randomly chosen MREs on the X or autosome 2L (columns 3 and 5). B) Heatmaps show the enrichment of CG1832 surrounding MREs from ChIP-seq data in SL2 cells. Enrichment of CG1832 is shown surrounding the same MREs that were examined in 4A. The log2 (ChIP/input) scale is used for binned read count; the enrichment difference is enhanced in the ChIP-seq data owing to its greater dynamic range. C) Profiles of genome-wide localization data are shown for CG1832 (red), MBD-R2 (blue), and Chromator (green), in a 30 Kb region including the *roX2* locus.

To define NSL complex and CG1832 localization on chromatin, we analyzed the genome-wide occupancy of both factors. Genome-wide localization data were available for the Chromator/Chriz and MBD-R2 components of the NSL complex (www.modENCODE.org). Therefore, we analyzed the enrichment of these proteins at CES loci compared with other sites that contain MRE sequences. Results indicate that there is a broad enrichment of NSL complex components within a 10 kb region surrounding CES compared to additional genomic loci on the X and autosomes that contain MREs (Figure 4A), consistent with a local or regional function.

To assess the genome-wide occupancy of CG1832, we generated a polyclonal antibody that targets the N-terminus of the protein (see Methods). We validated that the CG1832 antibody recognizes a protein of the predicted size and its RNAi knockdown strongly reduces its protein and mRNA levels and association with chromatin (Figures S1, S2, S3). CG1832 is expressed in both males and females and binds to polytene chromosomes in both sexes, consistent with it having a general, essential function in addition to its potential role in facilitation of MSL targeting (Figures S2, S4). We performed ChIP-seq analysis on the CG1832 protein in male SL2 cells and mapped 2,695 CG1832 sites on the X and 10,009 on autosomes using inclusive statistical criteria for defining binding sites (see Methods). Overall, MREs on the X chromosome are approximately 1.5-fold more likely to be occupied by CG1832 than MREs on autosomes (49.7% on X: 1832/3683; 32.5% on autosomes: 3787/11619). Although CG1832 occupancy is not X-specific, it is highly enriched at CES loci. In fact, 98.5% (135/137) of the genetically defined CES that contain MRE sequences [3] have a CG1832 binding site. Also, 92.5% (446/484) of all MSL complex binding sites that contain an MRE also have a CG1832 binding site. This expanded set would include likely CES not characterized previously. Most compelling, CG1832 occupancy at MREs is strongly enriched at CES compared with locations on the X and autosomes that contain MRE sequences but fail to recruit MSL complex, as visualized by heat maps for enrichment at various classes of MREs (Figure 4B). Comparison of ChIP-seq and ChIP-chip profiles of CG1832, MBD-R2, and Chromator show similar localization to 5′ ends of active genes, with CG1832 more focused at CES (Figure 4C). The precise pattern of enrichment over MREs makes CG1832 a strong candidate to directly recognize these sequences. These results demonstrate that our genetic screen has identified at least two new regulators of MSL complex recruitment that are implicated in functioning directly at chromatin entry sites.

## Discussion

Using a novel cell-based RNAi screening approach, we have identified new candidate regulators of MSL complex function. The screen took advantage of the ∼five fold regulation of a *roX2*-luciferase reporter construct by MSL complex, rather than relying on a direct assay of dosage compensation (<two fold) or on more laborious assays for MSL occupancy. Many of the regulators that we have identified are likely to have additional functions in both male and female flies and therefore could not have been recovered from classical male-specific lethal genetic screens. In addition to direct regulators of MSL complex recruitment, it is possible that transcriptional regulators identified from our screening approach could function indirectly to regulate transcription of MSL complex components themselves. Therefore, we used chromatin immunoprecipitation and existing modEncode data to demonstrate that two factors, the NSL complex and CG1832, are likely to have local roles in MSL complex recruitment to CES. Moreover, CG1832 is a strong candidate to recruit MSL complex directly to CES due to its strong enrichment at MRE sequences.

Interestingly, we identified a previously unstudied function of the highly-conserved NSL complex at CES loci. NSL and MSL complex share the MOF histone acetyltransferase as their catalytic component [13] [36] and there are conserved MOF protein-protein interaction interfaces between MSL1 and NSL1 [25]. Therefore, it is possible that MSL complex and NSL complex would compete for the MOF subunit, suggesting that NSL complex would antagonize MSL complex. However, we instead found that NSL complex facilitates MSL complex recruitment to CES (Figure 3). Therefore, it is possible that MOF may be recruited to MSL entry sites via NSL complex components such as MBD-R2 or RCD-5 that are known to be involved in MOF recruitment to 5′ ends of active genes [37] [36]. Subsequently, MOF could be transferred to newly assembling MSL complexes at chromatin entry sites as previously suggested [25]. Alternatively, since functional CES are enriched in the vicinity of active genes [38], this could indicate a role for NSL-dependent active gene expression in CES function.

Many additional activators were identified from our RNAi screening approach including all components of the PAF transcription complex that associates with RNAP II across gene bodies to promote transcription elongation and polyadenylation [26] [16]. However, RNAi treatment targeting the PAF complex did not alter MSL complex recruitment to entry sites or MSL target genes (Figure 3A). There are several possible mechanisms by which PAF complex could regulate our MSL-dependent reporter gene. The PAF complex has been implicated in facilitating H2B ubquitylation catalyzed by the *S. cerevisiae* SAGA complex component Ubp8 [27] [28]. H2B was also identified as an activator in our screen potentially implicating H2B ubiquitylation in facilitating MSL-dependent activity of the reporter (Table S1). Moreover, recent work has identified a new activity for the MSL2 protein as an E3 ligase that targets H2B for ubiquitylation [29]. Therefore, it is possible that the PAF complex facilitates the H2B ubiquitin ligase activity of MSL2. Alternatively, PAF has been implicated in regulating H3K36me3 [39] suggesting that it could also mediate MSL complex activity by modulating deposition of this known regulator of MSL complex recruitment to active genes.

In addition to identifying activators of MSL-dependent reporter activity, we identified several repressors including NURF301, a known repressor of MSL complex function at the *roX* CES loci [24]. In this way, we validated that additional candidates have potential to directly function in repressing MSL complex recruitment or activity. Other repressors of known function include the Sfmbt protein that recruits the Polycomb repressor complex and the large TRRAP protein that mediates interactions between gene specific regulators and large transcription complexes [40]. It is possible that these proteins and other potential repressors directly antagonize MSL complex function or alter the chromatin environment around CES to reduce transcriptional activation.

Our most exciting new candidate is CG1832, a previously unstudied zinc-finger protein that is strongly and precisely enriched at MRE sequences within CES and facilitates MSL complex recruitment to these sites. This protein has seven zinc-fingers at its C-terminus that could potentially bind DNA and a glutamine-rich N-terminus that may interact with MSL complex. Therefore, future analyses will focus on the exciting possibility that CG1832 functions as a previously unknown link between MSL complex and MRE sequences.

In summary, our novel screening approach identified two regulators that facilitate MSL complex recruitment at CES: NSL complex and CG1832. Furthermore, our genetic screen provides an extensive dataset of additional candidate genes that may facilitate MSL targeting and function. We expect that future genetic, genomic, and biochemical approaches will define new mechanisms by which many of these candidate genes modulate coordinate gene regulation.

## Methods

### Genome-wide RNAi screen

A genome-wide RNAi screen was performed in duplicate using the version 2 RNAi libraries generated by the Drosophila RNAi Screening Center (www.flyrnai.org) [41]. Transient transfection of MSL-dependent and independent luciferase reporter plasmids was conducted as described previously [3]. For screening, all volumes of plasmids and luciferase reagents were reduced by 4-fold to accommodate a 384-well format from the original 96-well format. As controls, GFP dsRNA was added to two wells and MSL2 dsRNA was added to two additional wells on every screening plate. GFP and MSL2 dsRNA was prepared as described previously [42]. All RNAi treatments were conducted for 5 days.

### Identification of candidate regulators of MSL complex targeting from RNAi screening data

First, the ratio of the Firefly luciferase to the Renilla luciferase activity was computed for each well in each of the 124 screening plates. Second, the Firefly/Renilla activity ratios for the GFP dsRNA wells and the MSL2 dsRNA wells were compared to assure that at least a 5-fold dynamic range was present for each plate. All plates fulfilled these criteria. Third, we calculated the median Firefly/Renilla ratio for each plate and its standard deviation. Our criterion for identifying a well as a positive screening hit was that its Firefly luciferase/Renilla luciferase ratio is more that two standard deviations above or below the plate median for both replicate plates. Most 384-well plates had 2–5 screening hits. To eliminate screening hits that cause non-specific cell lethality or low transfection efficiency, we did not consider wells with Renilla luciferase values that were more than three-fold below the plate median. Secondary screens using validation dsRNAs and additional plasmids described in [3] were performed using the same protocol and analysis approach. All dsRNA amplicons are identified in Table S1 by their DRSC number.

### mRNA analysis and Western blotting

mRNA analysis and qRT-PCR was performed after the following RNAi treatments: 1) DRSC03718 to target CG1832; 2) DRSC15625 to target NSL1 (CG4699); and 3) DRSC27502 to target PAF1 (*atms*). All RNAi treatments were conducted for 5 days in SL2 cells as previously described [42]. An additional CG1832 RNAi construct (DRSC 29935) was also tested with similar results (data not shown).

Western blotting was conducted as previously described using standard protocols [42]. We generated the rabbit polyclonal CG1832 antibody against part of the glutamine-rich domain of the protein (amino acids: #22-121) that was determined to be unique in the *Drosophila* genome. For Western blotting, the CG1832 antibody was used at a 1∶1000 dilution, the tubulin antibody (Sigma) was used at a 1∶10,000 dilution, and the histone H3 antibody (Abcam) was used at a 1∶1000 dilution.

### Chromatin immunoprecipitation

Chromatin immunoprecipitation from SL2 cells was conducted as described previously [42] using antibodies targeting the MSL2 protein, H4K16ac (Millipore), H3 (Abcam), and CG1832 (SDI) proteins. RNAi treatments were performed using the following DRSC RNAi constructs (www.flyrnai.org): 1) DRSC03718 to target CG1832; 2) DRSC15625 to target NSL1 (CG4699); and 3) DRSC27502 to target PAF1 (*atms*). To assure reproducibility, large scale RNAi experiments were performed in which 225 ug of each dsRNA was added to a T225 flask containing 45 mls of SL2 cells. An additional CG1832 RNAi construct (DRSC 29935) was also tested with similar results (data not shown).

Three independent chromatin preparations were performed for each experiment and qPCR was performed on input and IP samples using primers that are specific for MSL target genes and CES loci. Primer sequences were previously described [42]. Standard deviations among the three replicates were calculated after normalization to a sample that was treated with the GFP dsRNA construct as a control within each chromatin preparation. mRNA extraction followed by qRT-PCR was performed in parallel with each ChIP experiment to assure that the RNAi treatments were effective.

### Preparation of ChIP-seq libraries

Duplicate ChIP-seq libraries from independent chromatin preparations were generated using a protocol adapted from the Illumina ChIP-seq sample preparation guide as follows (www.illumina.com). To obtain sufficient starting material, three IPs were pooled and concentrated using Qiagen MinElute columns such that 15 ng of starting material were obtained prior to library preparation. Libraries from matching input samples were also prepared for each chromatin preparation. Each enzyme reaction was followed by purification using AMPure XP Beads (Agilent). The fragmented DNA was 3′ end repaired and 5′ phosphorylated using End-it Kit (Epicentre). A 3′ adenosine was added using Klenow Fragment (3′→5′ exo) (NEB). The annealed adapters were ligated in a 1∶50 dilution of the 10 mM stock using T4 DNA Ligase Quick Ligase Kit (Enzymatics). The libraries were PCR amplified using Phusion high fidelity polymerase (NEB) for 15 cycles. The libraries were then size selected on a 2% agarose gel using the E-Gel system (Invitrogen) selecting a range from 200 bp to 500 bp followed by concentration on a MinElute Column (Qiagen). Sequencing was performed on the Illumina GaIIx platform.

### Analysis of ChIP-seq data

Reads were aligned to the dm3 assembly of the *D. melanogaster* genome using the Bowtie aligner [43]. A summary of the sequencing statistics is provided below:

Replicate #1 input: 29495696 raw reads, 17591247 aligned reads (59.6%)

Replicate #1 CG1832 IP: 27820142 raw reads, 19210177 aligned reads (69.1%)

Replicate #2 input: 22498098 raw reads, 13510882 aligned reads (60.1%)

Replicate #2 CG1832 IP: 28231400 raw reads, 15194724 aligned reads (53.8%)

Pearson R value for CG1832 ChIP-seq replicates, 1 kb bins: 0.95

Reads aligned with more than one mismatch, and reads that did not align uniquely were discarded. Peaks were called in each ChIP-seq sample using the SPP software package with an FDR threshold of 0.05 [44]. For comparisons with MSL complex ChIP-seq data, MSL complex ChIP-seq data [3] were realigned with Bowtie and peaks were called with SPP in the same manner as the CG1832 ChIP-seq samples, for more direct comparison. The locations of CES loci and MRE sequences were previously reported [3]. Data will be available at NCBI GEO Short Read Archive (SRA) upon publication.

### Generation of heatmaps

The average enrichment profiles of proteins around the MREs (+/−5 kb) are shown in Figure 4. The five columns in the heatmap are the following: ‘MRE in CES’ consists of experimentally obtained 140 MREs (located within 137 CES) described in [3]; ‘Best MRE on X’ and ‘Best MRE on 2L’ consist of 150 MREs that have the best consensus motif match on the X or 2L, respectively; and ‘Random MRE on X’ and ‘Random MRE on 2L’ consist of 150 MREs randomly chosen from the X or 2L, respectively. More details can be found in Alekseyenko et al, 2012 (submitted).

### modENCODE ChIP-chip data processing

ChIP-chip data using Genomic DNA Tiling Arrays v2.0 (Affymetrix) are publicly accessible online through the modENCODE project (www.modencode.org). Data analysis was performed in R statistical programming environment (http://www.r-project.org). For the visualization of the heatmap (e.g., Figure 4), the +/−5 kb region surrounding each MRE was separated into non-overlapping bins of 200 bp. The smoothed probe value within each bin is averaged to obtain the enrichment value for that bin. Heatmaps were generated using the same classes of MRE containing loci as described above for the CG1832 ChIP-seq data.

## Supporting Information

Figure S1 mRNA levels of MSL complex components and candidates after CG1832, Nsl1, and Paf1 RNAi treatments. A) qRT-PCR was used to assay the expression levels of all MSL complex components present in SL2 cells. roX2 levels are decreased likely because targeting of MSL complex to the roX2 CES locus is altered by the RNAi treatments as expected. Expression levels of other MSL complex components are unchanged. All data were normalized to the control RNAi treatment and are the average of two replicates. B) Effective RNAi treatments were validated by qRT-PCR and the results shown are the average of two replicates.
(TIF)

Figure S2 Western blots for CG1832 RNAi treatment and antibody validation. A) Westerns on whole cell and nuclear extracts indicate that CG1832 is a nuclear protein and the CG1832 RNAi treatment reduces CG1832 protein levels. The arrow indicates the location of the CG1832 protein (60 kDa) and tubulin was used as a loading control. B) Nuclear extraction was performed on protein samples from SL2 (male) and Kc (female) cells followed by Western blotting. The histone H3 antibody was used as a loading control. Levels of CG1832 are similar in males and females.
(TIF)

Figure S3 Validation of CG1832 antibody by ChIP at CES loci. qPCR was performed on CG1832 ChIP samples before and after CG1832 RNAi treatment. Three independent CES loci were assayed and CG1832 RNAi strongly reduced the levels of CG1832 protein on chromatin at all three loci. An average of two independent experiments is shown.
(TIF)

Figure S4 Immunostaining of polytene chromosomes. Polytene staining using the CG1832 antibody (red) in male (A) and female (B) larvae. Co-staining was performed with an anti-MSL3 antibody (green). Co-localization of the two proteins is shown in yellow.
(TIF)

Table S1 Initial candidates identified from a genome-wide RNAi screen to identify novel regulators of dosage compensation.
(XLSX)

Table S2 Final candidates identified from a genome-wide RNAi screen. Candidates are color-coded and divided into functional categories described at the bottom of the table.
(PDF)
