# Supplementary figures and images for: Identification of Chromatin-Associated Regulators of MSL Complex Targeting in Drosophila Dosage Compensation
Source: PLoS Genet. 2012 Jul 26;8(7):e1002830. doi: 10.1371/journal.pgen.1002830 (PMC3405997; doi:10.1371/journal.pgen.1002830)

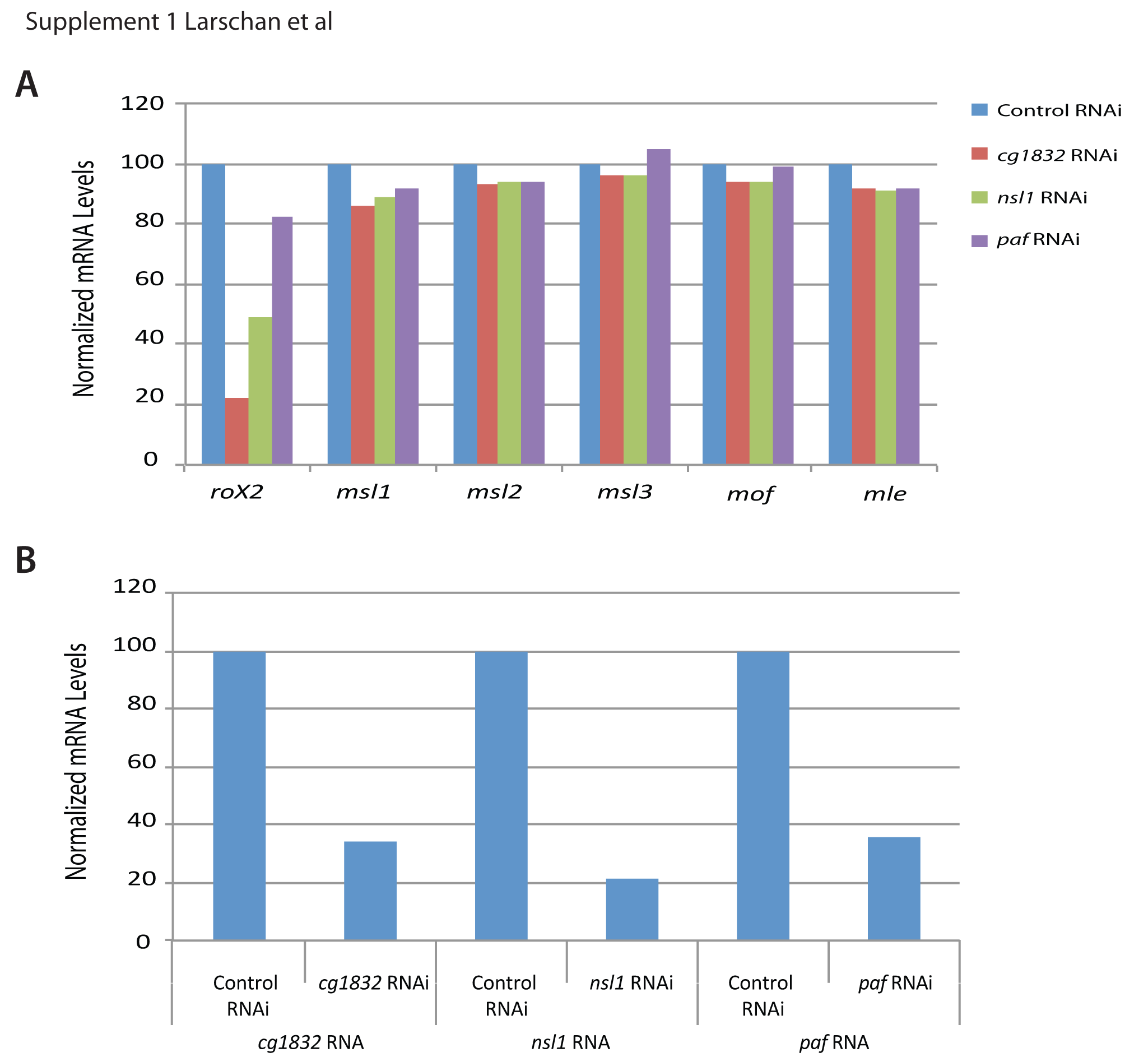

Supplement: Figure S1 — mRNA levels of MSL complex components and candidates after CG1832, Nsl1, and Paf1 RNAi treatments. A) qRT-PCR was used to assay the expression levels of all MSL complex components present in SL2 cells. roX2 levels are decreased likely because targeting of MSL complex to the roX2 CES locus is altered by the RNAi treatments as expected. Expression levels of other MSL complex components are unchanged. All data were normalized to the control RNAi treatment and are the average of two replicates. B) Effective RNAi treatments were validated by qRT-PCR and the results shown are the average of two replicates. (TIF) [file pgen.1002830.s001.tif]

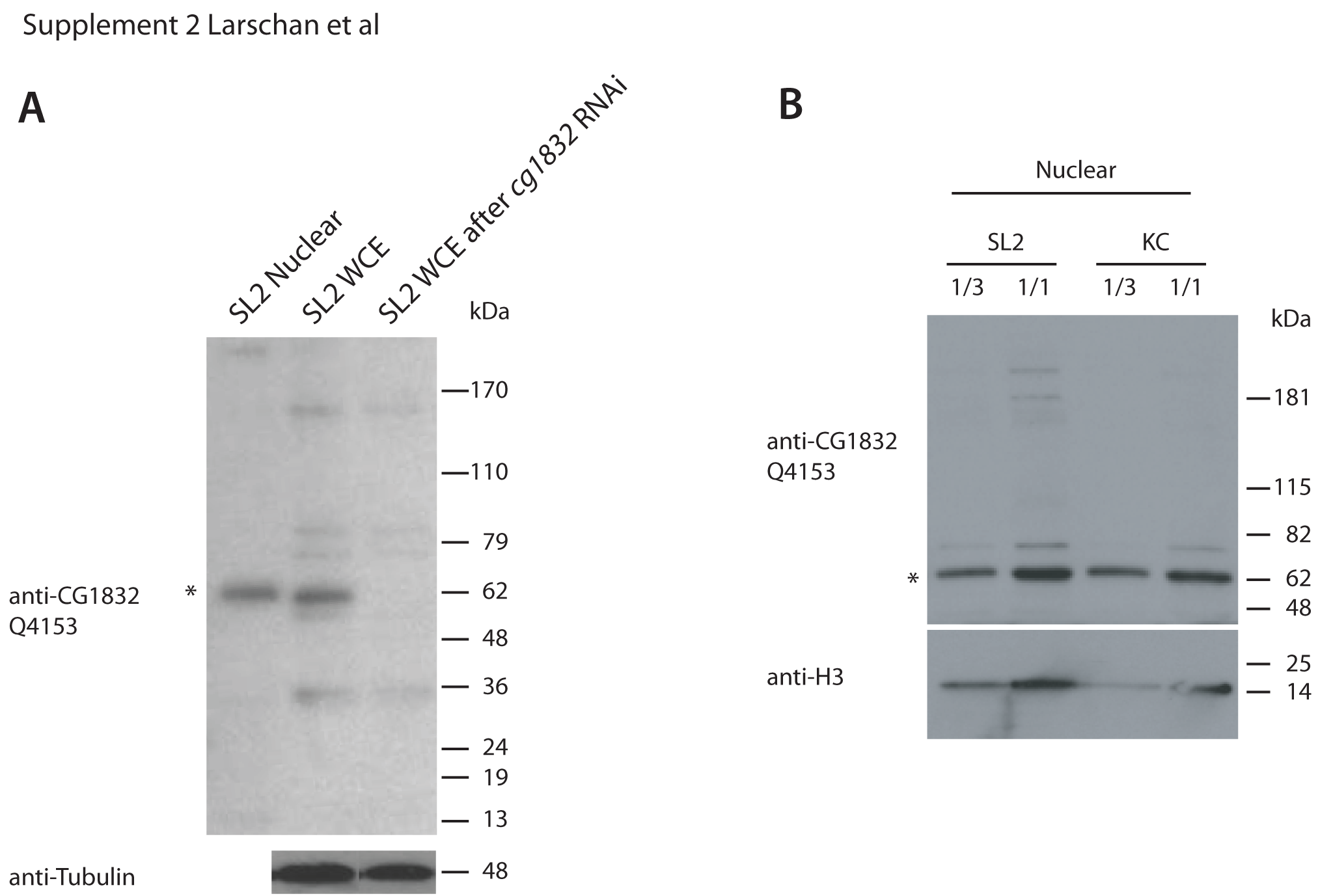

Supplement: Figure S2 — Western blots for CG1832 RNAi treatment and antibody validation. A) Westerns on whole cell and nuclear extracts indicate that CG1832 is a nuclear protein and the CG1832 RNAi treatment reduces CG1832 protein levels. The arrow indicates the location of the CG1832 protein (60 kDa) and tubulin was used as a loading control. B) Nuclear extraction was performed on protein samples from SL2 (male) and Kc (female) cells followed by Western blotting. The histone H3 antibody was used as a loading control. Levels of CG1832 are similar in males and females. (TIF) [file pgen.1002830.s002.tif]

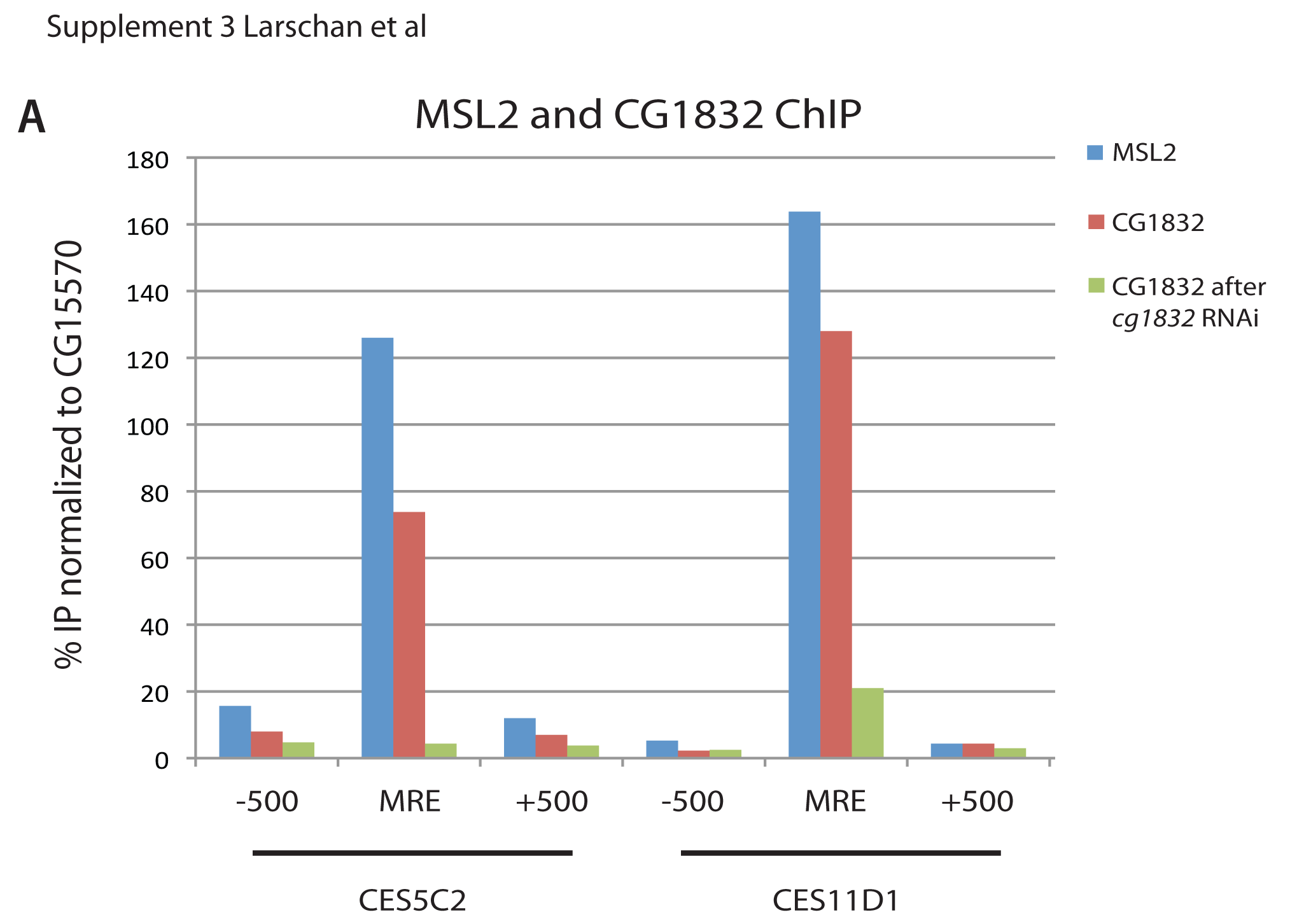

Supplement: Figure S3 — Validation of CG1832 antibody by ChIP at CES loci. qPCR was performed on CG1832 ChIP samples before and after CG1832 RNAi treatment. Three independent CES loci were assayed and CG1832 RNAi strongly reduced the levels of CG1832 protein on chromatin at all three loci. An average of two independent experiments is shown. (TIF) [file pgen.1002830.s003.tif]

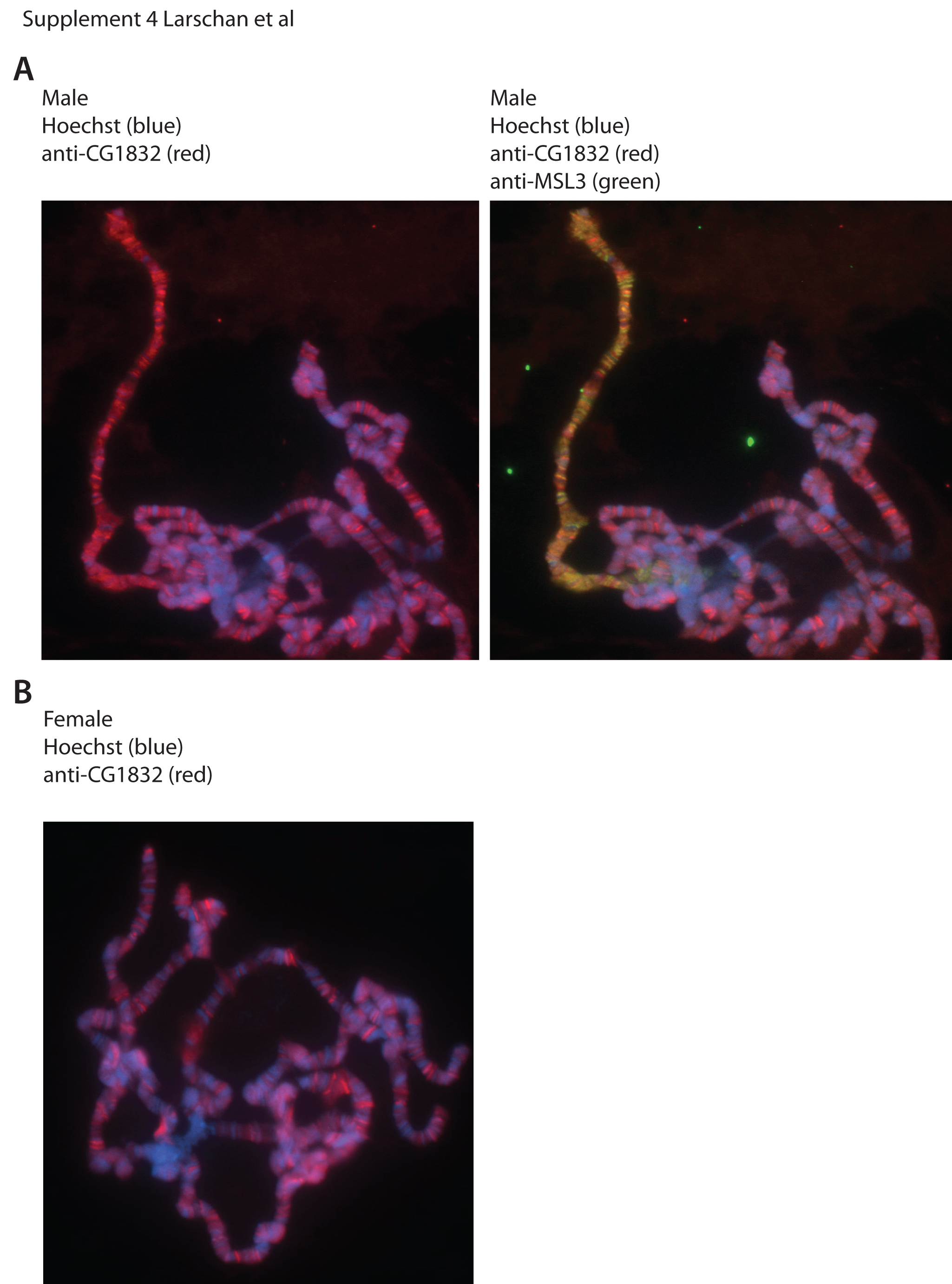

Supplement: Figure S4 — Immunostaining of polytene chromosomes. Polytene staining using the CG1832 antibody (red) in male (A) and female (B) larvae. Co-staining was performed with an anti-MSL3 antibody (green). Co-localization of the two proteins is shown in yellow. (TIF) [file pgen.1002830.s004.tif]
