# Supplementary material for: Identification of Chromatin-Associated Regulators of MSL Complex Targeting in Drosophila Dosage Compensation
Source: PLoS Genet. 2012 Jul 26;8(7):e1002830. doi: 10.1371/journal.pgen.1002830 (PMC3405997; doi:10.1371/journal.pgen.1002830)
Supplement: Table S2 — Final candidates identified from a genome-wide RNAi screen. Candidates are color-coded and divided into functional categories described at the bottom of the table. (PDF) [file pgen.1002830.s006.pdf]

Gene	Human homologue	Yeast Homologue	Firefly/Renilla	Description	Class
CG4699			0.17	NSL1	1
CG4756	SAP30L		0.24	Sin3associated protein	1
HDAC4	HDAC4		0.24	histone deacetylase	1
CG8233	FLJ10081		0.3	NSL3	1
CG1135	MCRS1		0.31	NSL component	1
Sin3A	SIN3A	SIN3	0.31	Sin3 corepressor	1
Dsp1	HMGB2		0.35	HMG box protein	1
MBD-R2			0.35	DNA binding TF	1
hyx	CDC73		0.36	paf component	1
CG2469	CTR9	CTR9	0.38	paf component	1
Z4			0.39	z4	1
CG14220	SUDS3		0.4	Sin3A complex member	1
atms	PAF1		0.41	Paf1	1
Trf2	TBPL1		0.46	TBP-like protein	1
His2B:CG3387	His2B:CG33870: HIST2	His2B:CG33870: HT	0.48	H2B	1
Atu	LEO1	LEO1	0.55	Leo1 Paf complex	1
Chro			0.6	chromatin architecture	1
trr			0.66	K4 methyltransferase	1
CG1832			0.25	transcription factor	2
Dp	TFDP2		0.3	Transcription factor related to E2F	2
Bx	LMO1		0.33	transcription factor	2
zfh1	ZEB1		0.39	zinc-finger transcription factor	2
D19A			0.52	transcription factor	2
D19B			0.52	transcription factor	2
nej	EP300		1.33	CBP/p300	3
E(bx)	BPTF		1.51	Nurf301	3
CG16975	L3MBTL2		1.73	transcription factor repressor	3
dom		SWR1	1.73	SWR1 ATP-dependent helicase	3
CG8211	INTS2		1.85	Integrator complex RNA Pol II associated 3' end formation	3
Nipped-A	TRRAP	TRA1	1.85	TRA1	3
caz	FUS		1.99	TFIID complex	3
srp			1.95	DNA-binding TF	4
lilli			2.03	DNA binding TF involved in neural development similar to AFF4	4
kay			2.31	DNA binding TF involved in neural development dpp pathway	4
hoip	NHP2L1	SNU13	0.25	mRNA splicing/export	5
Pcf11			0.29	mRNA cleavage factor	5
Cbp80	NCBP1		0.33	RNA cap-binding protein	5
Upf1	UPF1	NAM7	0.34	helicase involved in nonsense-mediated decay	5
Upf2	UPF2	NMD2	0.34	nonsense-mediated decay	5
Hel25E	BAT1	SUB2	0.43	Sub2 helicase involved in TREX complex	5
Smg1	SMG1		0.47	nonsense-mediated decay and microRNA function	5
gfzf			0.11	glutathione transferase activity	6
CycE	CCNE1		0.26	cyclin E	6
CG6015	CDC40		0.27	Splicing factor	6
CG8878			0.36	kinase activity	6
dup	CDT1		0.36	DNA replication factor	6
foi	SLC39A10		0.36	zinc ion transporter	6
dos			0.39	SH2/SH3 protein receptor	6
CG5514			0.41	putative cell wall component	6
puc	DUSP10		0.43	phosphatase involved in dorsal closure	6
raw			0.43	autophagic cell death	6
CG11451			0.44	cell communication	6
l(2)05070	PSMB6	PRE3	0.44	proteosome/endopeptidase	6
cdc2	CDC2		0.48	cdc2	6
cul-4	CUL4B		0.48	ubiquitin-ligase	6
CG10486			0.52	membrane cation transport	6
CG31258			0.52	No known function	6
CG32369			0.52	proteolysis	6
CG8636			0.52	mitotic spindle formation	6
eIF3-S9	EIF3S9	PRT1	0.54	translation initiation factor	6
dmt			0.57	nuclear nervous system development	6
eIF3-S10	EIF3S10	RPG1	0.64	translation initiation factor	6
CG31211	C6orf111		0.65	No known function	6
geminin			0.65	repressor of DNA replication	6
CG5146			0.66	No known function	6
Rho1	RHOC		1.62	GTPase activity	6
CG3363			2.86	No known function	6
msl-1			0.23	MSL1	MSL
msl-2			0.23	MSL2	MSL
msl-3	MSL3L1		0.39	MSL3	MSL
mle	DHX9		0.42	mle	MSL
mof	MYST1		0.52	mof	MSL

Class 1: General transcriptional regulators that activate
Class 2: Proteins that have potential specific DNA binding activity and activate our reporter
Class 3: General transcriptional regulators that repress our reporter
Class 4: Proteins that have potential specific DNA binding activity and repress our reporter
Class 5: Proteins that regulate RNA metabolism
Class 6: Proteins that are previously unstudied and/or have functions unrelated to transcription or RNA metabolism
